# Right ventricular and biatrial CMR strain analysis detects myocardial functional impairment after breast cancer therapy

**DOI:** 10.1007/s00330-025-12257-x

**Published:** 2025-12-19

**Authors:** Destina Gizem Aydemir, Isabel Molwitz, Antonia Beitzen-Heineke, Hang Chen, Mathias Meyer, Volkmar Müller, Gerhard Adam, Ersin Cavus, Enver Tahir, Jennifer Erley

**Affiliations:** 1 Department of Diagnostic and Interventional Radiology and Nuclear Medicine, University Medical Center Hamburg-Eppendorf, Hamburg, Germany; 2 II. Medical Clinic and Polyclinic, University Medical Center Hamburg-Eppendorf, Hamburg, Germany; 3 Department of Gynecology, University Medical Center Hamburg-Eppendorf, Hamburg, Germany; 4 Department of Cardiology, University Heart and Vascular Center Hamburg-Eppendorf, Hamburg, Germany

**Keywords:** Cardiovascular magnetic resonance, Feature tracking, Cancer therapy-related cardiac dysfunction, Breast cancer therapy, Anthracycline

## Abstract

**Objectives:**

To investigate breast cancer therapy-related myocardial cardiotoxicity by analyzing right ventricular (RV), left atrial (LA), and right atrial (RA) function using cardiovascular magnetic resonance feature tracking (CMR-FT).

**Materials and methods:**

This prospective single-center study involved 38 female breast cancer patients with a mean age of 50 ± 11 years, who received systemic anthracycline chemotherapy. CMR was performed in all patients before initiating therapy (at baseline (BL)) and after a 12-month follow-up (FU). FT was utilized to evaluate the RV global longitudinal (GLS), radial (GRS), and circumferential strain (GCS), as well as the biatrial reservoir, booster, and conduit strain. Paired *t*-tests were used to assess the differences in strain values from BL to FU.

**Results:**

The mean RV GLS of all patients was significantly attenuated at FU compared to BL (−25.2 ± 3.9% at BL vs −20.6 ± 4.4% at FU, *p* < 0.001), whereas RV GRS and GCS showed no significant change. LA reservoir strain was significantly reduced at FU (24.1 ± 3.2 at BL vs 22.7 ± 3.3% at FU, *p* = 0.005), while LA booster and conduit strain showed non-significant change. Similarly, RA reservoir strain decreased significantly from BL to FU (24.9 ± 3.3% at BL vs 23.2 ± 2.9% at FU, *p* = 0.019), whereas RA booster and conduit strain did not significantly change from BL to FU.

**Conclusions:**

Cancer therapy-related cardiac dysfunction (CTRCD) is not confined to the LV but affects the RV and the atria as well. Changes in RV GLS and LA/RA reservoir strain could serve as additional markers of subclinical cardiotoxicity in breast cancer patients.

**Key Points:**

***Question*** *The role of RV and biatrial strain in detecting CTRCD is unclear*.

***Findings*** *Biatrial reservoir strain and RV GLS were significantly attenuated after breast cancer therapy*.

***Clinical relevance*** *RV GLS and biatrial reservoir strain might serve as early markers of subclinical cardiotoxicity after breast cancer therapy and should thus be routinely determined in patients with suspected CTRCD*.

**Graphical Abstract:**

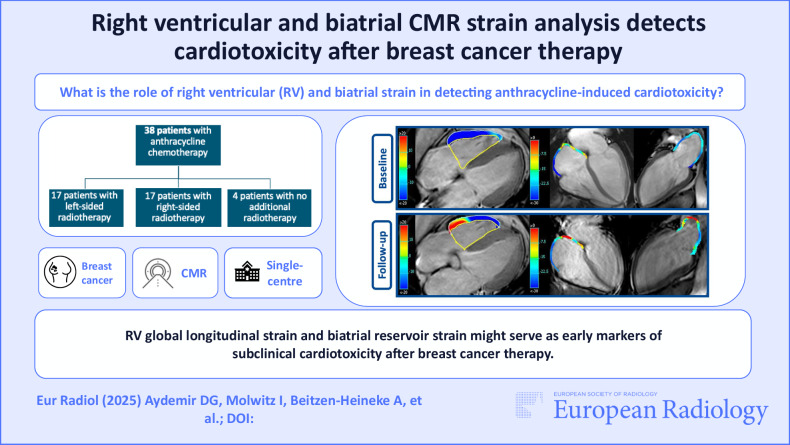

## Introduction

Female breast cancer is the most commonly diagnosed cancer worldwide, with an expected rise in incidence and mortality to 3 million new cases and 1 million deaths by 2040 [1]. Despite the use of alternative regimens, anthracyclines still play an important role in the treatment of breast cancer [2]. Adjuvant anthracycline-based chemotherapy regimens have been shown to drastically reduce the risk of disease recurrence, as well as the mortality in breast cancer patients [3]. However, anthracyclines may lead to irreversible cardiotoxicity, subsumed under the term “cancer therapy-related cardiac dysfunction” (CTRCD), which limits their therapeutic benefit [4]. The incidence of CTRCD following anthracycline chemotherapy reaches up to 67% with doxorubicin, whereas epirubicin is associated with a lower incidence of around 20% [5–8]. Early detection of CTRCD during or after anthracycline chemotherapy is important to prevent irreversible cardiac damage, manifesting as cardiomyopathy and heart failure, which contributes to further morbidity and mortality in breast cancer patients [9, 10]. Additionally, radiotherapy has been reported to induce cardiac toxicity in certain cases, although the current evidence regarding this association remains controversial [11–14].

Myocardial strain is a powerful tool to evaluate the regional and global deformation of the myocardium [15]. Ventricular strain parameters include global longitudinal (GLS), circumferential (GCS), and radial strain (GRS). The reservoir (εs), conduit (εe), and booster (εa) strains are used to measure atrial deformation. Although the ejection fraction (EF) is a commonly used parameter to evaluate left ventricular (LV) function, LV GLS has been shown to detect early subclinical cardiotoxicity, even when the LVEF remains preserved [16, 17]. A relative change in LV GLS exceeding 15%, and/or a reduction in LVEF, indicates CTRCD, whereas a normal LVEF does not exclude CTRCD according to current guidelines [18, 19].

Indeed, current European Society of Cardiology (ESC) guidelines recommend cardiac risk stratification before starting anthracycline-based chemotherapy and radiotherapy, to prevent adverse cardiac outcomes during and after the treatment [18]. Echocardiography is the first-line diagnostic imaging modality for the baseline (BL) assessment of cardiac function in cancer patients, whereas cardiac magnetic resonance (CMR) imaging is considered the gold standard for assessing cardiac function, with high reproducibility [20, 21]. Moreover, CMR is superior to echocardiography regarding strain quantification as the latter has shown a suboptimal field of view, higher interobserver variability, and lower spatial resolution [22]. Using CMR, feature tracking (FT) is a common method for myocardial strain analysis. While LV GLS is a well-established parameter to detect subclinical cardiotoxicity, there is limited data on the impact of anthracyclines on RV and biatrial function [23, 24]. Atrial strain, consisting of the reservoir (εs), conduit (εe), and booster strain (εa), is known to precede changes in ventricular function and serves as a predictor of mortality in various cardiac diseases, such as heart failure with preserved EF, as well as ischemic heart disease, and cardiomyopathies [25, 26]. Therefore, the purpose of this study was to evaluate longitudinal changes in RV and biatrial function in breast cancer patients using CMR-FT.

## Materials and methods

The study was approved by the local ethics committee (PV5292) and complied with local ethical guidelines [8]. This is a post-hoc analysis of a prospective, longitudinal study. Data on cardiac volumetric analysis, left ventricular function/strain, as well as multiparametric mapping sequences in this patient cohort have been previously published by Tahir et al [8].

### Study population

Patients with breast cancer received anthracycline-based (epirubicin) chemotherapy for 5 ± 1 months, followed by radiotherapy (RT), at the University Medical Center Hamburg-Eppendorf between October 2016 and October 2018. The chemotherapy regimen consisted of four cycles of epirubicin (90 mg/m²) in combination with cyclophosphamide (600 mg/m²), followed by 12 cycles of paclitaxel (80 mg/m²) according to guidelines [2]. Furthermore, five patients with human epidermal growth factor receptor-positive (HER2+) status also received sequential trastuzumab. CMR imaging was performed prior to therapy (BL) and 13 ± 2 months after BL (follow-up (FU)).

### CMR imaging

Details on the CMR protocol can be found in the previously published work by Tahir et al [8]. To summarize, CMR imaging was conducted using a 3 T scanner (*Ingenia, Philips Medical Systems*), including balanced steady-state free-precession (SSFP) cine images in a short-axis stack (SAX), and 2-, 3-, and 4-chamber (CH) long-axis views for strain quantification using FT. The sequence protocol is listed in Table 1.

**Table 1:** CMR sequence protocol

Component	Sequence/method	Key imaging parameters
Cine CMR	SSFP	AVS: 2 × 2 × 8 mm³; RVS: 0.99 × 0.99 × 8 mm³; no gap; 9–10 slices; TE: 1.45 ms; TR: 2.90 ms; flip angle: 45°; SENSE factor: 2
T1 mapping	MOLLI (5 s[3 s]3 s)	Voxel size: 2 × 2 × 10 mm³; TE: 0.7 ms; TR: 2.3 ms; flip angle: 35°; SENSE: 2; TI range: 134–5627 ms
T2 mapping	GraSE	Voxel size: 2 × 2 × 8 mm³; 3 slices; 9 echoes (TE: 10.7–96.3 ms); TR: 800 ms; 1 breath-hold per slice
LGE imaging	PSIR	AVS: 1.6 × 1.9 × 8 mm³; RVS: 0.91 × 0.91 × 8 mm³; gap: 2 mm; 9–10 slices; TE: 3 ms; TR: 6.10 ms; flip angle: 25°

*AVS* acquired voxel size, *GraSE* gradient- and spin-echo, *LGE* late gadolinium enhancement, *MOLLI* modified look-locker inversion recovery, *PSIR* phase-sensitive inversion recovery, *RVS* reconstructed voxel size, *SENSE* sensitivity encoding, *SSFP* steady-state free precession, *TE* echo time, *TI* inversion time, *TR* repetition time

### CMR post-processing

**Fig. 1:**
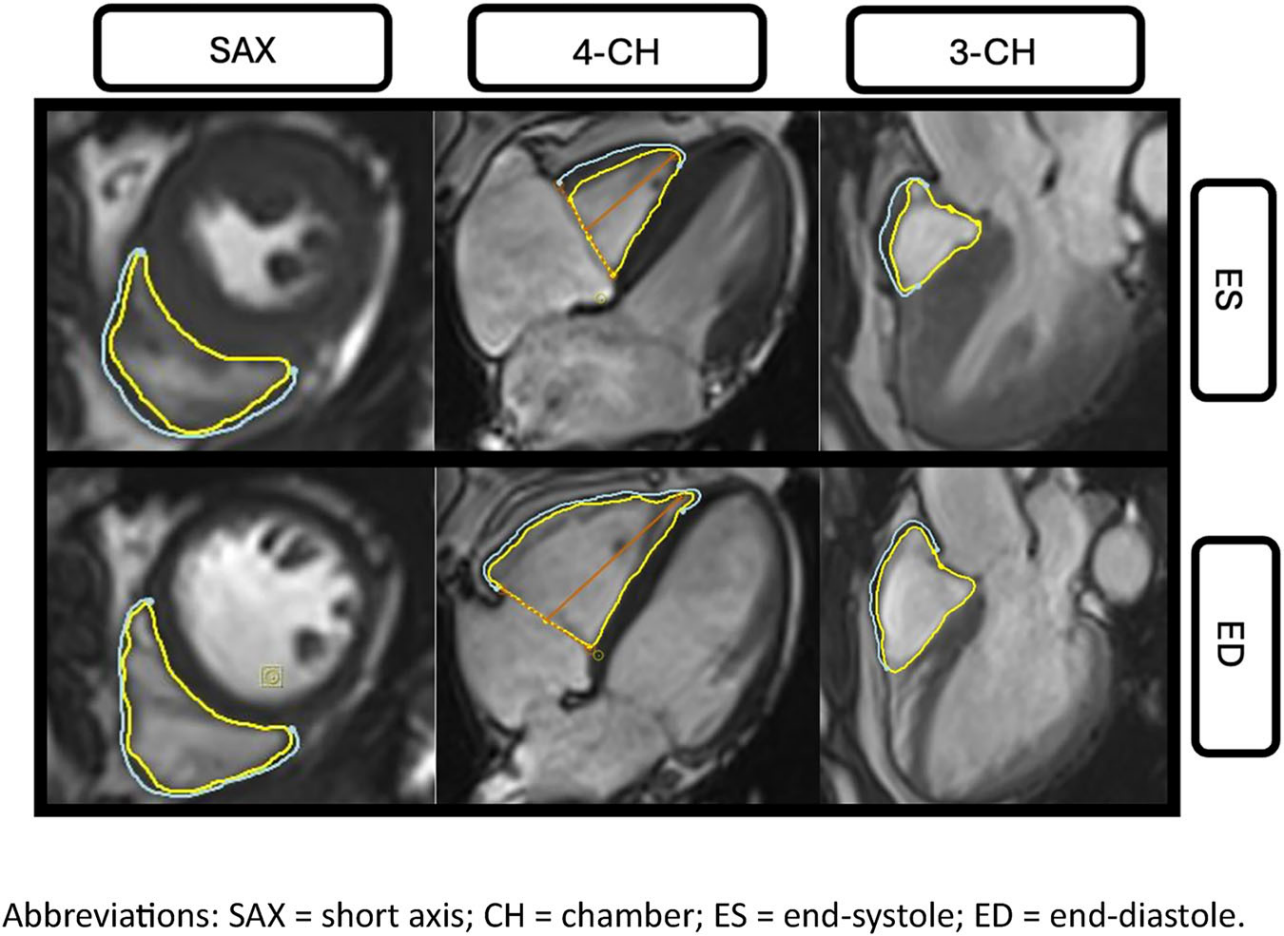
Manually drawn endocardial (yellow) and epicardial (blue) contours of the RV in SAX, 4- and 3-CH view at ES and ED for strain analysis. SAX, short axis; CH, chamber; ES, end-systole; ED, end-diastole

The FT module of the cvi^42^ (*Circle Cardiovascular Imaging Inc., Calgary, Canada*) software was used to assess RV, right atrial (RA), and left atrial (LA) myocardial strain. RV strain analysis consisted of GLS, GRS, and GCS. RV epi-and endocardial borders were manually contoured in the SAX stack at end-diastole (ED) and end-systole (ES) (Fig. 1). Likewise, epi- and endocardial contours were drawn in the 3- and 4-CH views for quantification of GLS. The septum was excluded from RV strain measurements, and care was taken to exclude papillary muscles and trabeculae from the contours.

**Fig. 2:**
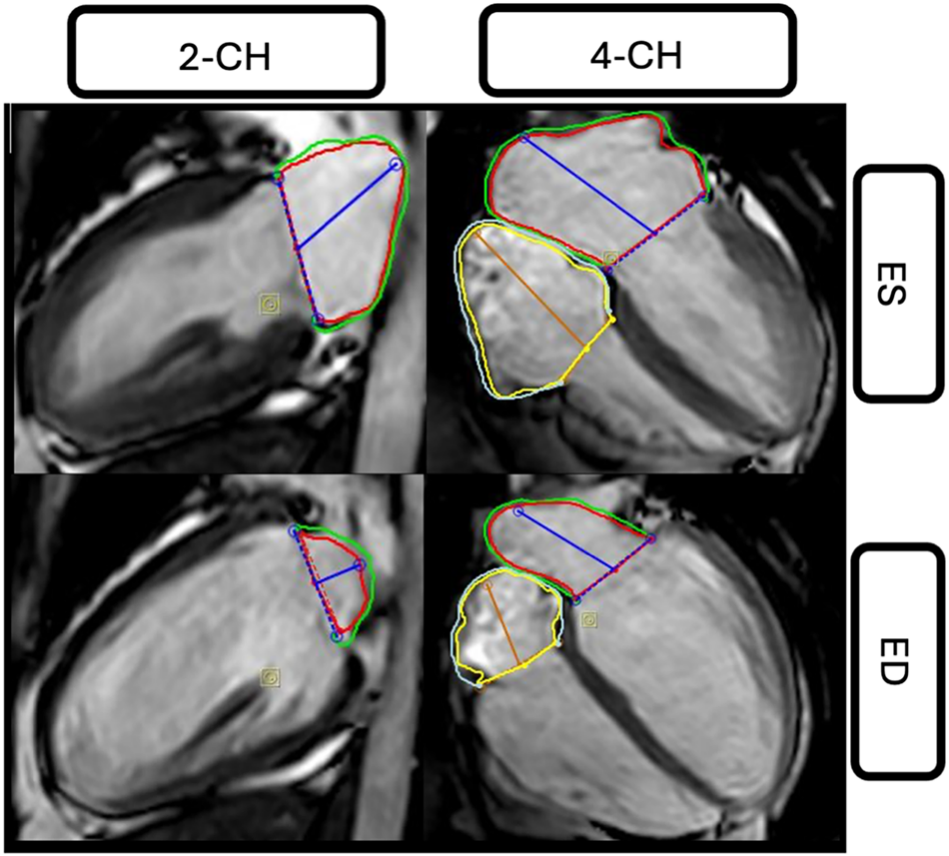
LA endocardial (red) and epicardial (green) contours in 2-CH and 4-CH views, as well as RA endocardial (yellow) and epicardial (blue) contours in the 4-CH view, were manually drawn at ES and ED. CH, chamber; ES, end-systole; ED, end-diastole; LA, left atrium; RA, right atrium

Biatrial strain parameters included the reservoir (εs), conduit (εe), and booster (εa) longitudinal strain, which are derived from the three main functions of the atria during the cardiac cycle: The reservoir function represents atrial filling during ventricular systole, leading to an increase in strain with positive values due to atrial expansion [27]. The conduit function corresponds to a passive atrial emptying in the early diastole, causing a negative strain [27]. The booster pump function refers to the atrial contraction during late diastole, also resulting in a negative strain [28]. For atrial strain analysis, epi- and endocardial contours were manually drawn in 4-CH (LA and RA) and 2-CH long-axis view (LA) at ED and ES (Fig. 2). Myocardial strain was analyzed by two radiologists with 4 and 5 years of experience in cardiac imaging. An intraclass correlation coefficient (ICC) was estimated to assess the interobserver agreement.

### Statistical analysis

Statistical analyses were conducted using RStudio (Version 2024.04.2 + 764, Posit Software, PBC). The Shapiro–Wilk test was used to evaluate normal distribution for all measurements. Normally distributed data are expressed as mean ± standard deviation (SD), and non-normally distributed data are described using median [interquartile range]. Differences between BL and FU CMR strain analyses were assessed using paired *t*-tests for normally distributed data and the Wilcoxon test for not normally distributed data. *t*-tests were used to assess the differences between independent groups. Analysis of variance (ANOVA) was performed for subgroup analyses. Moreover, differences in strain measurements between patients who received left- vs right-sided RT were assessed using Welch’s *t*-tests. To account for multiplicity, *p*-values were adjusted using the Benjamini–Hochberg false discovery rate (FDR, *q* < 0.05). Effect sizes were calculated as Cohen’s dz for paired comparisons (BL vs FU) and as Hedges’ g for independent comparisons (left-sided RT vs right-sided RT and RT vs no RT). *p*-values are considered descriptive due to the observational design of the study.

## Results

### Patient demographics

The current analysis includes 38 newly diagnosed breast cancer patients who underwent first-line anthracycline-based (epirubicin) chemotherapy and had BL and FU CMR imaging available. The age of the 38 breast cancer patients ranged from 26 to 75 years, with a mean age of 50 ± 12 years. One patient had known coronary artery disease, but showed a normal RVEF (54%), no regional wall motion abnormalities, and normal strain values at BL CMR. Otherwise, no patient had known cardiovascular disease. The detailed BL demographics, as well as the volumetric analysis and left ventricular strain results of the patients, can be found in a previously published manuscript by Tahir et al [8]. The mean cumulative dose of epirubicin was 661.7 ± 60.1 mg/m^2^. The mean cumulative dose of cyclophosphamide reached up to 4412 ± 400.1 mg/m^2^, and the mean dose of paclitaxel was 1673 ± 263.3 mg/m^2^. Of the 38 patients, 34 received additional RT (*n* = 17 left-sided, *n* = 17 right-sided). The flow chart of the study population can be found in Supplementary Material.

### Change in RV and biatrial strain values from BL to FU

**Fig. 3:**
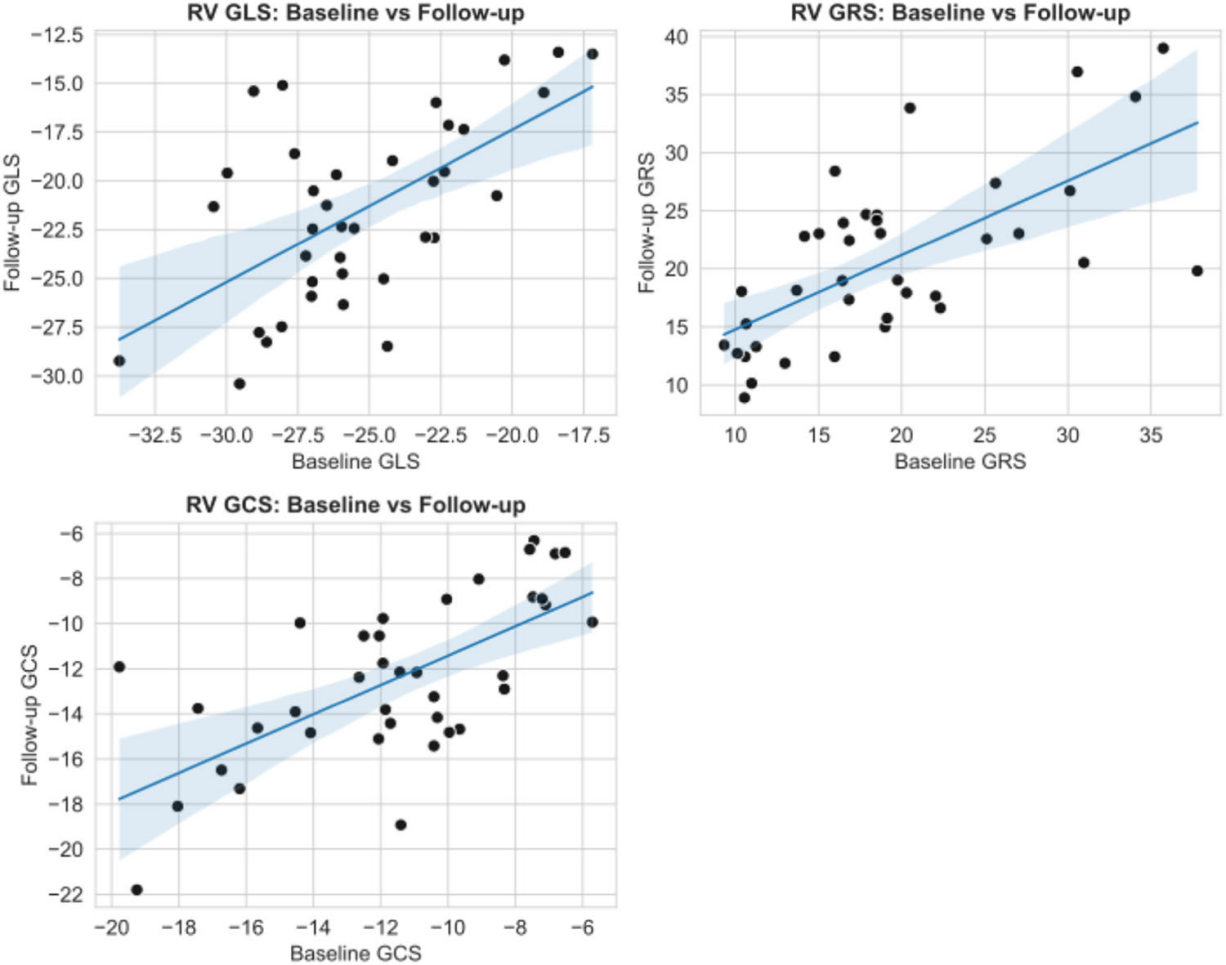
Scatterplots of RV GLS, GRS, and GCS comparing BL and FU measurements. Each point represents one patient. The blue line indicates the linear regression fit with a 95% confidence interval. RV, right ventricle; GCS, global circumferent strain; GLS, global longitudinal strain; GRS, global radial strain; BL, baseline; FU, follow-up

**Fig. 4:**
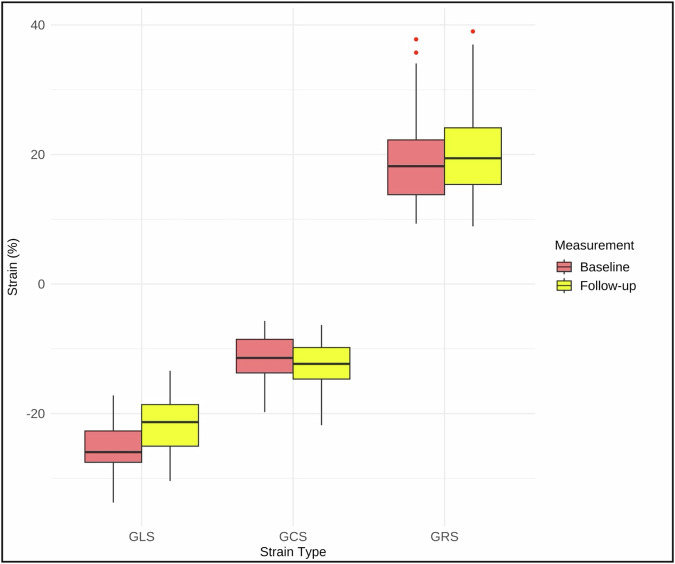
Box plots to display the attenuation in RV GLS, GCS, and GRS from BL to FU. Legend: The box plots visualize the significant increase in RV GLS, whereas the RV GRS and GCS show no significant changes at FU compared to BL. BL, baseline; FU, follow-up; GLS, global longitudinal strain; GCS, global circumferential strain; GRS, global radial strain; RV, right ventricle

**Fig. 5:**
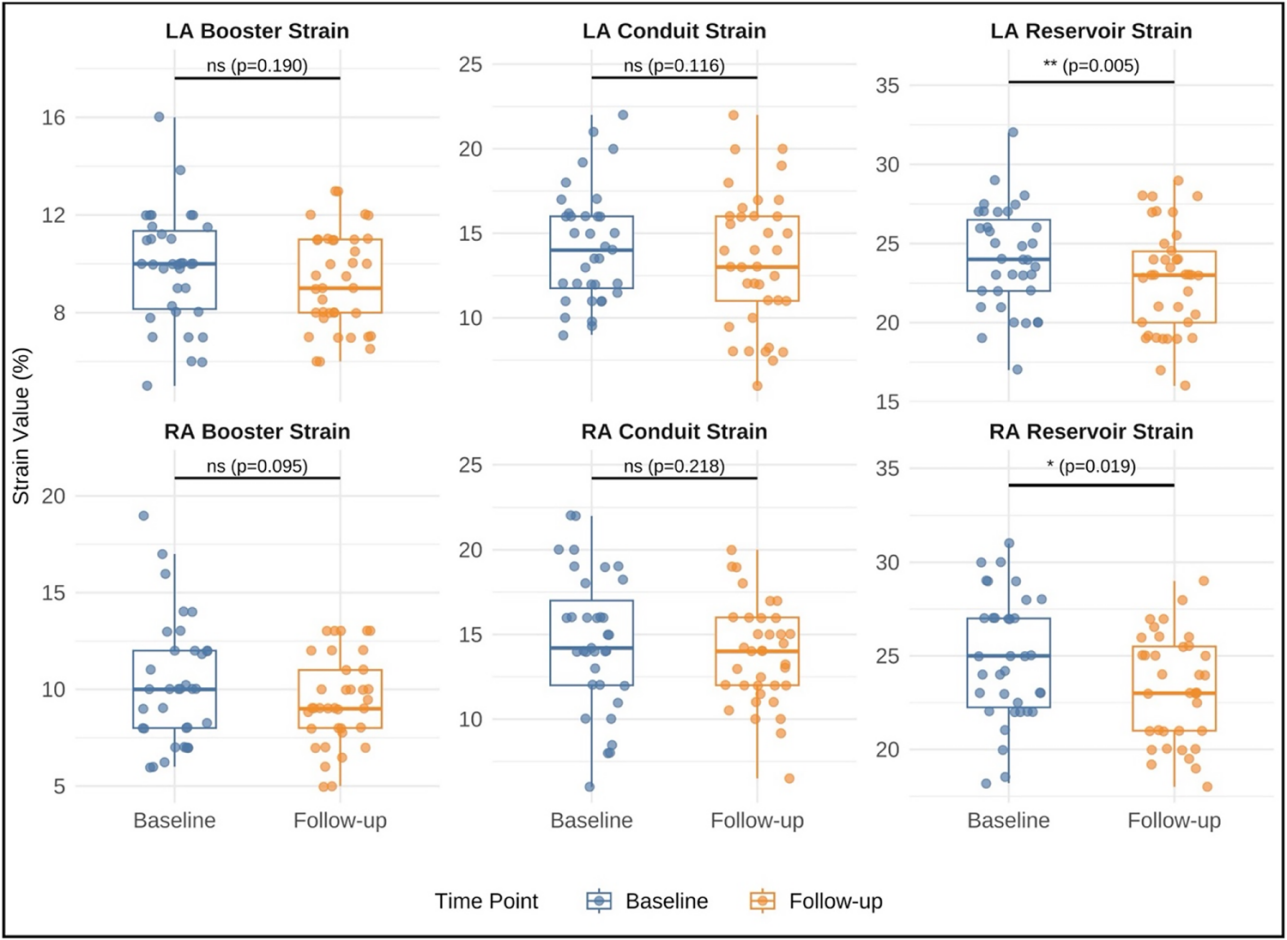
Box plots to display the LA and RA strain at BL and FU: The LA and RA reservoir strain was decreased at FU compared to BL, while the biatrial booster and conduit strain did not significantly change. LA, left atrium; RA, right atrium; BL, baseline; FU, follow-up; ns, not significant

**Fig. 6:**
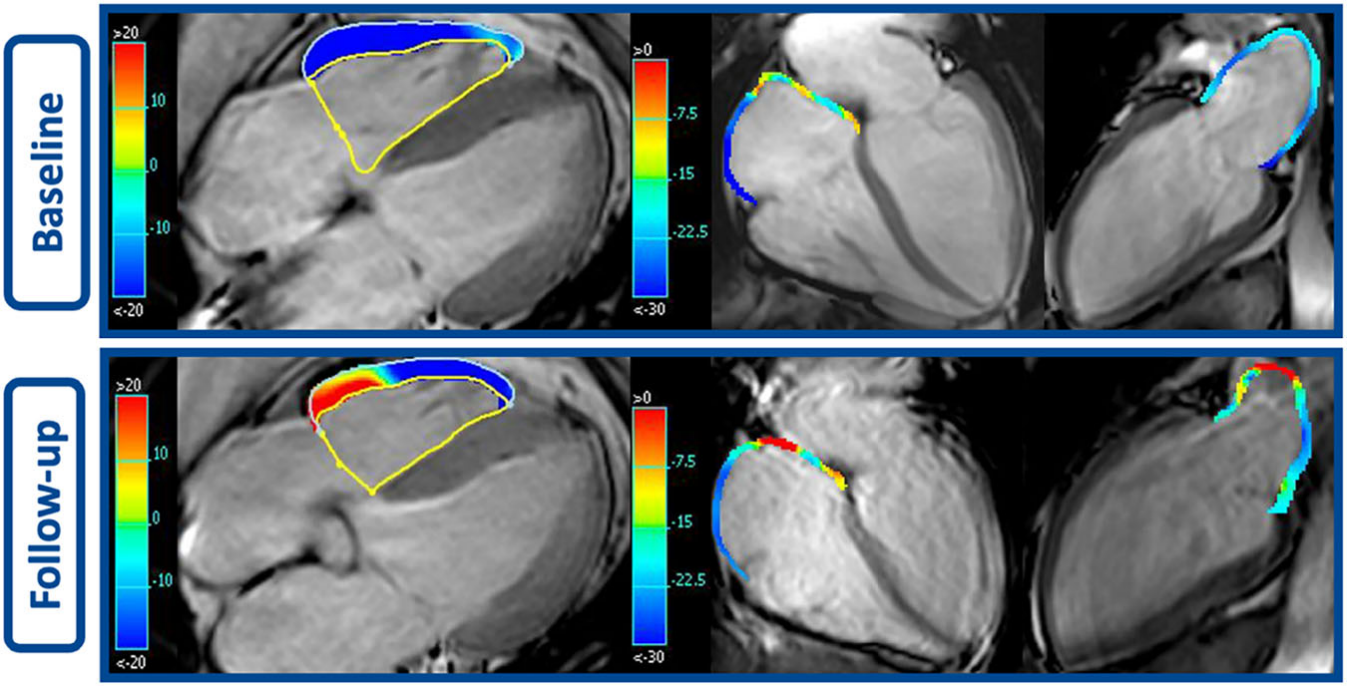
Exemplary cine-images at ED during post-processing using FT in a 61-year-old breast cancer patient at BL and FU to compare the RV GLS, as well as the biatrial reservoir strain. The color-coded overlay highlights the increase in RV GLS and the decrease in biatrial reservoir after chemotherapy, indicating reduced RV and biatrial contractility. BL, baseline; ED, end-diastole; FT, feature tracking; FU, follow-up; GLS, global longitudinal strain; RV, right ventricle

Three patients were excluded from the atrial strain analysis due to poor imaging quality. No patients were excluded from the RV strain analysis. The mean RV, LA, and RA strain values at BL and FU are shown in Table 2. The scatterplots demonstrate the RV strain parameters at BL and FU (Fig. 3). The mean RV GLS of all patients increased significantly from −25.2 ± 3.9% at BL to −20.6 ± 4.4% at FU (*p* < 0.001), indicating a reduction in RV contractility (Figure). The RV GRS (19 ± 7.4% vs 20.2 ± 6.8%, *p* = 0.099) and GCS (−11.4 ± 3.6% vs −12.1 ± 3.3%, *p* = 0.069) did not significantly change from BL to FU (Fig. 4). Figure 5 demonstrates the changes in biatrial strain between BL and FU. The LA reservoir strain decreased significantly from 24.1 ± 3.2% to 22.7 ± 3.3% (*p* = 0.005). The LA conduit strain showed no significant change (14.2 ± 3.3% vs 13.4 ± 4.0%, *p* = 0.117). Similarly, the LA booster strain did not significantly change from BL to FU (9.9 ± 2.3% vs 9.3 ± 2.0%, *p* = 0.190). The RA reservoir strain decreased significantly from 24.9 ± 3.3% to 23.2 ± 2.9% (*p* = 0.019) while the RA conduit strain showed no significant change (14.6 ± 4.0% vs 13.8 ± 3.0%, *p* = 0.218). Likewise, the RA booster strain did not significantly change from BL to FU (10 ± 3.2% to 9.4 ± 2.3%, *p* = 0.095). Figure 6 shows exemplary cine-images during post-processing with FT in a patient with increased RV GLS and decreased biatrial reservoir strain at FU compared to BL. After adjustment for multiple testing using the FDR, the changes in RV GLS and biatrial reservoir strain remained statistically significant (RV GLS: *p* < 0.01, *q* < 0.001; LA reservoir: *p* < 0.01, *q* = 0.015; RA reservoir: *p* < 0.05, *q* = 0.038). Post hoc power analysis indicated adequate power for LA reservoir strain (dz ≈ 0.51, power ≈ 0.83) and very high power for RV GLS (dz ≈ 0.98, power > 0.99), supporting the robustness of these findings. In contrast, findings regarding the RA reservoir strain only showed moderate power (dz ≈ 0.42, power ≈ 0.67).

**Table 2:** Mean strain values at BL and FU

Parameter	BL	FU	*p*-value
RV GLS	−25.3 ± 3.6	−21.5 ± 4.7	< 0.001
RV GRS	19.3 ± 7.6	20.7 ± 7.3	0.152
RV GCS	−11.5 ± 3.7	−12.4 ± 3.6	0.078
LA reservoir strain	24.1 ± 3.2	22.5 ± 3.3	0.005
LA conduit strain	14.2 ± 3.3	13.3 ± 4.1	0.116
LA booster strain	9.9 ± 2.3	9.2 ± 2.0	0.190
RA reservoir strain	24.9 ± 3.3	23.2 ± 2.9	0.019
RA conduit strain	14.6 ± 4.0	13.8 ± 3.0	0.218
RA booster strain	10.2 ± 3.2	9.3 ± 2.2	0.095

Numbers are mean ± standard deviation (SD) for continuous data. *p*-values from paired *t*-test, comparing differences between BL and FU

*BL* baseline, *FU* follow-up, *LA* left atrium, *RA* right atrium, *RT* radiotherapy, *RV* right ventricle, *GCS* global circumferential strain, *GLS* global longitudinal strain, *GRS* global radial strain

### Differences in RV and biatrial strain analyses between patients with and without LV CTRCD

As previously published by Tahir et al, 8 out of 38 patients developed CTRCD at FU, which is officially defined as LV dysfunction (decline in LVEF by ≥ 10% to < 55% or increase in LV GLS of ≥ 15%) (labeled as LV CTRCD in this analysis) [29]. Interestingly, 17 patients (45%) showed an increase in RV GLS of ≥ 15% from BL to FU. A reduction in LA and RA reservoir strain of ≥ 15% was observed in 13 (34%) and 11 patients (29%), respectively. Only 3 out of 8 patients with LV CTRCD showed a reduction in RV GLS of ≥ 15% at FU compared to BL (from −22.9 ± 4.1% to −18.0 ± 3.9%, *p* = 0.011). However, out of the 30 patients without the diagnosis of LV CTRCD, 14 patients also showed a ≥ 15% increase in RV GLS (from −25.1 ± 4.4% to −17.9 ± 4.0%, *p* < 0.001). The mean RV GLS increased from −25.6 ± 3.7% at BL to −21.7 ± 5.1% at FU in the non-LV CTRCD group (*p* < 0.001) and from −23.8 ± 3.1% to −20.9% ± 3.4% in the LV CTRCD group, respectively (*p* = 0.011). This change in RV GLS was not significantly different between the non-LV CTRCD and the LV CTRCD group (*p* = 0.448).

The mean LA reservoir strain showed a non-significant change from 24.4 ± 2.9% at BL to 23.2 ± 3.0% at FU in the non-LV CTRCD group (*p* = 0.123) but significantly decreased from 23.7 ± 4.4% at BL to 20 ± 2.5% at FU in the LV CTRCD group (*p* = 0.005), respectively. This decrease in LA reservoir strain was significantly stronger in the LV CTRCD group than in the non-LV CTRCD group according to the ANOVA analysis (*p* = 0.015). The mean RA reservoir strain decreased from 25.2 ± 3.4% at BL to 23.4 ± 3.0% at FU in the non-LV CTRCD group (*p* = 0.013) and from 23.5 ± 3.3% to 22.9 ± 2.9% in the LV CTRCD group (*p* = 0.403). This overall change in RA reservoir strain did not significantly differ between the LV CTRCD and non-LV CTRCD group (*p* = 0.363). Post hoc power for comparisons between these subgroups was uniformly low (RV GLS: 0.06, LA reservoir strain; 0.05, RA reservoir strain: 0.05). A decrease in LA and RA reservoir strain of ≥15% was seen in 7 patients (88%) and in 1 patient (13%) with LV CTRCD, respectively. Among patients without LV CTRCD, 5 (17%) showed a ≥ 15% decrease in LA reservoir strain, and 10 (33%) showed a ≥ 15% decrease in RA reservoir strain.

### Differences in RV and biatrial strain parameters between patients with and without additional RT

According to their RT status, patients were separated into three different groups: left-sided RT, right-sided RT, and no RT. A summary of the changes in RV, LA, and RA strain values between BL and FU across the RT groups can be found in Table 3. There were no significant differences in RV GLS between patients without RT (*n* = 4, −28.3 ± 0.6% at BL and −22.4 ± 6.4% at FU), with left-sided RT (*n* = 17, −24.4 ± 3.9% at BL vs −21.3 ± 4.4% at FU), and with right-sided RT (*n* = 17, −23.3 ± 3.8 at BL vs −21.6 ± 4.9% at FU; *p* = 0.293 at BL and *p* = 0.177 at FU). Similarly, there were no significant differences in RV GRS, RV GCS, and biatrial strain between the groups (all *p* > 0.05). However, the post hoc power for the RT subgroup comparisons was also very low (RV GLS: 0.06, LA reservoir: 0.05, RA reservoir: 0.05).

**Table 3:** Changes in RV and biatrial strain from BL to FU across radiotherapy groups

Parameter	Radiotherapy group	BL	FU	Difference	*p*-value
LA booster strain	No RT	10.60 ± 1.22	9.00 ± 0.87	−1.60 ± 2.08	0.252
	Left-sided RT	10.08 ± 2.68	8.79 ± 1.98	−1.28 ± 3.11	
	Right-sided RT	9.44 ± 2.02	9.72 ± 2.19	0.28 ± 2.49	
LA conduit strain	No RT	13.73 ± 3.29	14.17 ± 5.97	0.43 ± 3.30	0.298
	Left-sided RT	14.28 ± 3.39	13.98 ± 3.75	−0.29 ± 3.33	
	Right-sided RT	14.23 ± 3.52	12.30 ± 4.11	−1.93 ± 3.52	
LA reservoir strain	No RT	24.33 ± 3.79	23.17 ± 5.39	−1.17 ± 1.61	0.852
	Left-sided RT	24.35 ± 2.89	22.78 ± 3.30	−1.58 ± 2.60	
	Right-sided RT	23.67 ± 3.68	22.15 ± 3.11	−1.52 ± 3.68	
RA booster strain	No RT	13.00 ± 1.00	10.67 ± 2.08	−2.33 ± 1.15	0.670
	Left-sided RT	9.85 ± 3.05	8.90 ± 2.31	−0.95 ± 3.76	
	Right-sided RT	10.13 ± 3.48	9.42 ± 2.14	−0.71 ± 3.19	
RA conduit strain	No RT	14.33 ± 2.89	15.67 ± 3.51	1.33 ± 1.53	0.556
	Left-sided RT	14.32 ± 3.42	13.58 ± 3.44	−0.74 ± 3.64	
	Right-sided RT	15.03 ± 4.88	11.71 ± 2.45	−1.32 ± 4.30	
RA reservoir strain	No RT	27.33 ± 2.08	26.33 ± 2.31	−1.00 ± 1.73	0.719
	Left-sided RT	24.17 ± 3.57	22.54 ± 2.97	−1.63 ± 4.34	
	Right-sided RT	25.17 ± 3.10	23.27 ± 2.56	−1.90 ± 4.23	
RV GCS	No RT	−11.85 ± 4.51	−12.88 ± 4.36	−1.02 ± 2.94	0.642
	Left-sided RT	−11.18 ± 3.57	−11.86 ± 3.79	−0.68 ± 3.24	
	Right-sided RT	−11.85 ± 3.84	−12.89 ± 3.40	−1.04 ± 2.90	
RV GLS	No RT	−28.34 ± 0.62	−22.44 ± 6.41	5.90 ± 6.55	0.497
	Left-sided RT	−24.58 ± 3.65	−21.29 ± 4.41	3.30 ± 2.93	
	Right-sided RT	−25.31 ± 3.79	−21.58 ± 4.91	3.74 ± 4.01	
RV GRS	No RT	18.95 ± 8.59	23.46 ± 11.29	4.52 ± 6.64	0.529
	Left-sided RT	18.82 ± 7.33	19.76 ± 7.46	0.95 ± 6.20	
	Right-sided RT	19.78 ± 8.17	21.00 ± 6.43	1.23 ± 6.05	

Numbers are mean ± standard deviation (SD). *p*-values from ANOVA or Kruskal-Wallis test, comparing differences across radiotherapy groups

*LA* left atrium, *RA* right atrium, *RT* radiotherapy, *RV* right ventricle, *GCS* global circumferential strain, *GLS* global longitudinal strain, *GRS* global radial strain

### Interobserver reproducibility

Detailed results on interobserver reproducibility are presented in Table 4. RV strain parameters showed good reproducibility, with ICC values ranging from 0.80 (RV GLS) to 0.88 (RV GCS). Similarly, atrial strain analysis demonstrated good to excellent interobserver agreement, with ICC values between 0.76 (RA booster strain) and 0.92 (RA conduit strain).

**Table 4:** ICC for the RV and biatrial strain analysis to show interobserver agreement

Parameter	ICC	95% CI
RV GLS	0.80	[0.47, 0.93]
RV GRS	0.81	[0.49, 0.94]
RV GCS	0.88	[0.65, 0.96]
LA reservoir strain	0.84	[0.58, 0.95]
LA conduit strain	0.88	[0.66, 0.96]
LA booster strain	0.90	[0.72, 0.97]
RA reservoir strain	0.85	[0.59, 0.95]
RA conduit strain	0.92	[0.75, 0.97]
RA booster strain	0.76	[0.39, 0.92]

*LA* left atrium, *RA* right atrium, *RV* right ventricle, *GCS* global circumferential strain, *GLS* global longitudinal strain, *GRS* global radial strain

## Discussion

This study investigated the changes in RV, LA, and RA strain after breast cancer therapy via CMR-FT.

The major findings of our study are as follows:

1. RV GLS, as well as biatrial reservoir strain, showed a significant attenuation at 12 months after breast cancer therapy, whereas RV GRS, RV GCS, biatrial booster, and conduit strain showed no significant changes.
2. LA reservoir strain was significantly reduced at FU in patients with LV CTRCD compared to patients without LV CTRCD, while changes in RV GLS and RA reservoir strain were similar between the groups with and without LV CTRCD.
3. Relative changes of ≥ 15% in RV GLS, as well as biatrial reservoir strain, were more frequent than the occurrence of LV CTRCD.

As the gold standard for myocardial tissue characterization and functional assessment, CMR offers superior and reproducible four-chamber strain analysis compared to other imaging modalities, including echocardiography [30]. Using CMR-FT, we noticed a significant increase in RV GLS from BL to FU CMR after breast cancer therapy, including anthracycline-based chemotherapy. These findings are in line with a previous analysis of Rossetto et al, who also reported a significant increase in LV and RV GLS following anthracycline-based chemotherapy in a similar patient cohort, thereby reinforcing the existing evidence [24]. The increase in RV GLS noticed in this study (from −25.2 ± 3.9% at BL to −20.6 ± 4.4% at FU, *p* < 0.001) was more pronounced than the increase in LV GLS reported by Tahir et al in the same patient cohort (from −18 ± 2% at BL to −17 ± 2% at FU, *p* < 0.05) [8]. In this cohort, 17 patients exhibited a relative increase in RV GLS of ≥ 15%, while only 8 patients showed a comparable increase in LV GLS, as published by Tahir et al [8]. Therefore, the current CTRCD criteria were fulfilled more frequently for the RV GLS than for the LV GLS. Similar findings were previously reported by Laufer-Perl et al, who also found a more frequent increase in RV GLS following anthracycline-based chemotherapy than in LV GLS [31]. Notably, the increase in RV GLS in this patient group was not linked to the current official diagnosis of CTRCD, which is defined by LV function. In fact, 15 patients in this study demonstrated a ≥ 15% relative increase in RV GLS at FU despite maintaining normal LV-function. A possible reason for this finding could be, that the RV might be more sensitive to anthracycline-induced toxicity than the LV, which may be attributed to its thin wall composed predominantly of longitudinal fibers. These longitudinal fibers appear more vulnerable to anthracycline-induced toxicity [32] than the circumferential and radial myocardial fibers. Further prospective studies are needed to investigate, whether the effect of breast cancer therapy is stronger on the RV than on the LV, and if functional RV measurements might, therefore, be a more sensitive marker of subclinical cardiotoxicity.

Furthermore, we observed a significant decrease in LA reservoir strain at FU in this study, while booster and conduit strain did not significantly change. These findings are consistent with previous results by Di Lisi et al and Mokadem et al, who also reported reduced LA reservoir strain after anthracycline chemotherapy [23, 33]. The decrease in LA reservoir strain was significantly higher in patients with CTRCD [8]. In subgroup analyses, a ≥ 15% relative decrease in LA reservoir strain was more frequently observed in patients with LV CTRCD than without, whereas the decrease in RA reservoir strain was comparable across these groups. Di Lisi et al also reported a greater decline in LA strain after anthracycline chemotherapy in patients with CTRCD compared to patients without CTRCD, supporting the connection between LV and LA dysfunction [23]. However, post hoc power for the CTRCD subgroup comparisons was low, which limits the strength of non-significant findings.

To our knowledge, this is the first study to investigate the effect of anthracycline therapy on RA function. In parallel to the LA, we observed a significant reduction in RA reservoir strain at FU, whereas RA conduit and booster strain remained stable. These changes in ventricular and biatrial strain could be caused by anthracycline-induced oxidative stress, which leads to mitochondrial damage and apoptosis in both ventricular and atrial cardiomyocytes and fibroblasts, triggering myocardial fibrosis and remodeling [34]. The resulting reduction in atrial compliance and elasticity likely explains why reservoir strain is affected first, as this phase reflects atrial filling during ventricular systole, which diminishes early in the course of myocardial injury caused by anthracycline-based chemotherapy [35]. As previous studies have demonstrated a strong association between reduced atrial strain and increased mortality in multiple cardiovascular conditions, further research is warranted to determine whether alterations in biatrial strain similarly predict overall mortality and could refine patient risk stratification [36–38].

To our knowledge, this is also the first study to investigate the effect of radiotherapy on biatrial function in breast cancer patients receiving anthracycline-based chemotherapy. Previous research by Trivedi et al reported a significant increase in LV GLS between breast cancer patients with and without left-sided RT [13]. Another study by Honaryar et al also showed a significant increase in LV GLS, based on 2D-speckle echocardiography in breast cancer patients after RT and without anthracycline-based chemotherapy [11]. However, like Tahir et al did not report any differences in LV strain between patients with and without RT in this cohort [8], we also could not find any significant difference in RV and biatrial strain across the RT groups. These results align with those of Hassan et al and Marrazzo et al, who likewise did not demonstrate a significant association between RT and changes in LV GLS [12, 14]. However, the post hoc power of these analyses was low, reflecting the limited number of patients without RT and underscoring that the statistically nonsignificant findings should be interpreted with caution. Further prospective studies with larger patient cohorts are needed to fully differentiate between the cardiac effects of chemotherapy alone and those potentially resulting from combined chemo- and radiotherapy.

Our study had several important limitations that should be considered. The primary limitation, as mentioned above, is the relatively small cohort size, which may have affected the statistical power to detect subtle differences between groups. A further limitation is the absence of long-term clinical outcome data, such as the incidence of heart failure or overall survival. Consequently, the present findings should provide preliminary insights that require confirmation in larger prospective cohorts with long-term clinical follow-up. Another important limitation of our study was the limited number of patients without RT after chemotherapy, which limited our ability to clearly differentiate between cardiac changes caused by chemotherapy vs those potentially influenced by the combination of radiotherapy and chemotherapy. The heterogeneity in treatment protocols, as well as the different cumulative doses of chemotherapy agents among the patients, are further limitations of this study. Future prospective studies with larger, more homogenous patient cohorts would help confirm our findings and potentially identify additional associations between cancer therapy and cardiac outcomes, especially since the clinical significance of the subclinical changes remains unclear.

In conclusion, the RV GLS, as well as the RA and LA reservoir strain, could serve as early markers of myocardial damage after breast cancer therapy, as is already known for the LV GLS. RV GLS and biatrial reservoir strain appeared more often pathological than LV GLS when applying the ≥ 15% relative change threshold for CTRCD, suggesting their importance as early markers of potential cardiotoxic effects. The implementation of RV and atrial strain analyses into the routine FU of breast cancer patients could therefore facilitate the early detection of cardiotoxicity, which may eventually lead to global heart failure.

## Supplementary information

ELECTRONIC SUPPLEMENTARY MATERIAL

## Abbreviations

BL: Baseline
CH: Chamber
CMR: Cardiac magnetic resonance
CTRCD: Cancer therapy-related cardiac dysfunction
EF: Ejection fraction
FDR: False discovery rate
FT: Feature tracking
FU: Follow-up
GCS: Global circumferential strain
GLS: Global longitudinal strain
GRS: Global radial strain
ICC: Intraclass correlation coefficient
LA: Left atrium
LV: Left ventricle
RA: Right atrium
RV: Right ventricle
SAX: Short axis
SSFP: Standard steady-state free-precession
