# Supplementary material for: Right ventricular and biatrial CMR strain analysis detects myocardial functional impairment after breast cancer therapy
Source: Eur Radiol. 2025 Dec 19;36(5):4037–48. doi: 10.1007/s00330-025-12257-x (PMC13086657; doi:10.1007/s00330-025-12257-x)
Supplement: Supplementary file 1 — ELECTRONIC SUPPLEMENTARY MATERIAL [file 330_2025_12257_MOESM1_ESM.pdf]

Right ventricular and biatrial CMR strain analysis detects myocardial functional impairment after breast cancer therapy

ELECTRONIC SUPPLEMENTARY MATERIAL

Flow chart of the study population

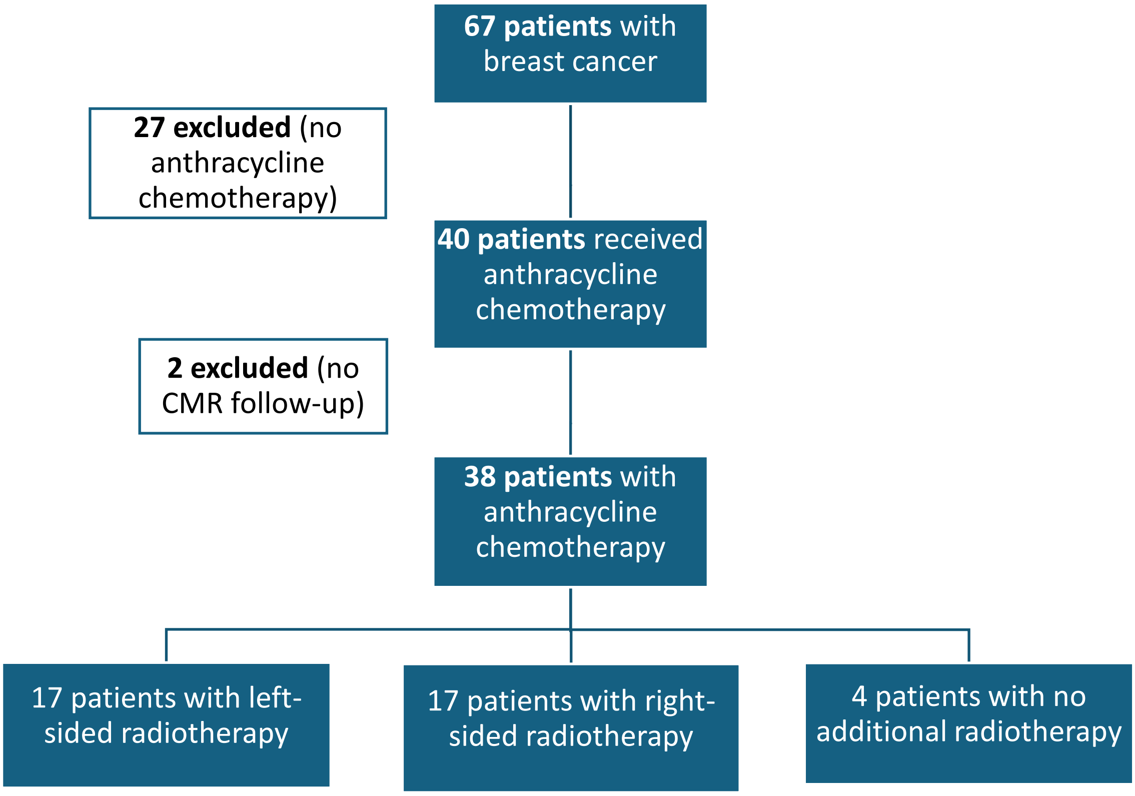

Eur Radiol (2025) Aydemir DG, Molwitz I, Beitzen-Heineke A, et al.
